# Molecular Characterization of *Corynebacterium diphtheriae* Outbreak Isolates, South Africa, March–June 2015

**DOI:** 10.3201/eid2308.162039

**Published:** 2017-08

**Authors:** Mignon du Plessis, Nicole Wolter, Mushal Allam, Linda de Gouveia, Fahima Moosa, Genevie Ntshoe, Lucille Blumberg, Cheryl Cohen, Marshagne Smith, Portia Mutevedzi, Juno Thomas, Valentino Horne, Prashini Moodley, Moherndran Archary, Yesholata Mahabeer, Saajida Mahomed, Warren Kuhn, Koleka Mlisana, Kerrigan McCarthy, Anne von Gottberg

**Affiliations:** National Health Laboratory Service, Johannesburg, South Africa (M. du Plessis, N. Wolter, M. Allam, L. de Gouveia, F. Moosa, G. Ntshoe, L. Blumberg, C. Cohen, M. Smith, P. Mutevedzi, J. Thomas, K. McCarthy, A. von Gottberg);; University of the Witwatersrand, Johannesburg (M. du Plessis, N. Wolter, C. Cohen, A. von Gottberg);; National Health Laboratory Service, Cape Town, South Africa (V. Horne);; University of KwaZulu-Natal, Durban, South Africa (P. Moodley, M. Archary, Y. Mahabeer, S. Mahomed, W. Kuhn, K. Mlisana);; National Health Laboratory Service, Durban (Y. Mahabeer, K. Mlisana);; National Department of Health, KwaZulu-Natal, South Africa (W. Kuhn)

**Keywords:** *Corynebacterium diphtheriae*, outbreak, South Africa, diphtheria, MLST, whole-genome sequencing, molecular epidemiology, respiratory diphtheria, cutaneous diphtheria, sequence type, CRISPR, bacteria, respiratory infections

## Abstract

In 2015, a cluster of respiratory diphtheria cases was reported from KwaZulu-Natal Province in South Africa. By using whole-genome analysis, we characterized 21 *Corynebacterium diphtheriae* isolates collected from 20 patients and contacts during the outbreak (1 patient was infected with 2 variants of *C. diphtheriae*). In addition, we included 1 cutaneous isolate, 2 endocarditis isolates, and 2 archived clinical isolates (ca. 1980) for comparison. Two novel lineages were identified, namely, toxigenic sequence type (ST) ST-378 (n = 17) and nontoxigenic ST-395 (n = 3). One archived isolate and the cutaneous isolate were ST-395, suggesting ongoing circulation of this lineage for >30 years. The absence of preexisting molecular sequence data limits drawing conclusions pertaining to the origin of these strains; however, these findings provide baseline genotypic data for future cases and outbreaks. Neither ST has been reported in any other country; this ST appears to be endemic only in South Africa.

Respiratory diphtheria, caused by toxigenic strains of the gram-positive bacillus *Corynebacterium diphtheriae*, is an upper respiratory tract disease characterized by a sore throat; mild fever; and gray-white pseudomembrane on the tonsils, larynx, or pharynx. Introduction of the diphtheria toxoid vaccine in 1923 and widespread mass immunization in the 1940s and 1950s led to the near elimination of the disease in the industrialized world (*1*). However, diphtheria remains endemic in many developing countries despite implementation of the World Health Organization Expanded Programme on Immunization in 1974. Epidemic diphtheria resurged in Russia and Eastern Europe in the early 1990s, with >150,000 reported cases occurring predominantly in older children and adults (*2*).

Molecular epidemiology can be used for investigating diphtheria case clusters in the postvaccine era to improve our understanding of patterns of transmission and spread of epidemic clones. At the time of the Russia epidemic, ribotyping was the established gold standard method of strain typing; however, if deviations from the prescribed protocols occurred, reproducibility could have been compromised (*3*). Subsequently, multilocus sequence typing (MLST) was developed, which is highly reproducible and provides more accurate information regarding the population structure and evolution (*4*). More recently, core-genome phylogenetic analyses showed a high level of discrimination and was able to provide insight into *C. diphtheriae* genomic diversity and identify factors contributing to virulence (*5*). In addition, the highly discriminatory, clustered, regularly interspaced short palindromic repeats (CRISPR) spoligotyping was used for a more detailed analysis of the Russia epidemic clone (*6*).

In South Africa, early studies in the 1940s and 1950s reported rates of respiratory diphtheria significantly higher than those in industrialized countries, ranging 20–35 cases/100,000 population and equating to ≈3,000 case notifications annually (*7*,*8*). During 1980–2014, a total of 412 diphtheria cases were reported in South Africa through the World Health Organization–UNICEF joint reporting process with most (>80%) notified before 1990 (*9*). The last laboratory-confirmed respiratory diphtheria case reported in South Africa occurred in a 22-year-old woman in February 2010 in Western Cape Province (*10*).

During March–June 2015, a cluster of 15 respiratory diphtheria patients with a case-fatality ratio of 27% was reported from KwaZulu-Natal Province in South Africa (*11*). In 2014, before the outbreak, a KwaZulu-Natal official reported that the province had 96% coverage for the primary series of diphtheria vaccinations and 83% coverage for the 18-month booster (N. McKerrow, KwaZulu-Natal Department of Health, pers. comm., 2015 Jun 8). However, the tetanus-diphtheria booster coverage rates were 54% for 6-year-olds and 20% for 12-year-olds. In response to the outbreak of diphtheria, contact tracing was conducted and postexposure prophylaxis was given to family members and school and clinic contacts (*11*). Educational leaflets about social mobilization and health promotion activities were distributed in affected communities. The KwaZulu-Natal Department of Health embarked on a catch-up vaccination campaign for schoolgoing children 6–15 years of age in the affected districts. National guidelines for the management and public health response to diphtheria were developed (*12*), and healthcare practitioners countrywide were notified to be on the alert for possible cases. Laboratories in South Africa were requested to include selective media for isolation of *C. diphtheriae* when processing throat swabs and to submit all potential *C. diphtheriae* isolates to the national reference laboratory for confirmation and to establish an isolate repository for molecular surveillance. We conducted a molecular epidemiologic investigation by using whole-genome data to characterize isolates from cases and contacts linked to this KwaZulu-Natal outbreak.

## Methods

### Definitions

We defined a confirmed case as the occurrence of clinical symptoms consistent with respiratory diphtheria (sore throat; low-grade fever; and an adherent membrane on the pharynx, tonsils, larynx, or nose) in a person who was positive for toxin-producing *C. diphtheriae* and a probable case as the occurrence of mild respiratory symptoms or clinical symptoms of respiratory diphtheria in a *C. diphtheriae* culture–negative person who was epidemiologically linked to a patient or carrier positive for toxin-producing *C. diphtheriae*. For the purposes of this investigation, we defined a carrier as a person with a laboratory-confirmed, toxin-producing or non–toxin-producing *C. diphtheriae* infection with no respiratory symptoms.

### Bacterial Strain Collection

During the 2015 KwaZulu-Natal diphtheria outbreak investigation, we received 21 *C. diphtheriae* isolates swabbed from the throat or nasopharynx (or groin in 1 case). We confirmed identification of cultures by matrix-assisted laser desorption/ionization time-of-flight technology (*13*) and confirmed the presence of the A and B subunit genes of the *C. diphtheriae* toxin and phenotypic toxin production by 2 different real-time PCR assays (*14*,*15*) and Elek testing (*16*). Isolates were biotyped with the API Coryne kit (BioMérieux, Lyon, France). We included 2 archived clinical isolates of *C. diphtheriae* that were isolated in South Africa during the 1980s (although no clinical or demographic data were available for these isolates) and 2 *C. diphtheriae* isolates from preadolescent children with endocarditis obtained in July and August 2015 (Table 1). Positive controls for PCR and Elek testing were *C. diphtheriae* vaccine-type strain PW8 (ATCC 13812; American Type Culture Collection, Manassas, VA, USA); toxin-positive *C. diphtheriae* NCTC 3984 and 10648 (National Collection of Type Cultures, Salisbury, UK); and toxin-negative *C. diphtheriae* NCTC 10356. Negative controls were *C. ulcerans* (NCTC 12077), *C. bovis* (ATCC 7715), and *C. striatum* (ATCC BAA-1293) (Table 1).

**Table 1:** *Corynebacterium* controls and nonoutbreak–associated *C. diphtheriae* isolates from South Africa*

Isolate no.	Organism (biotype)	Toxin-producing	ST
ATCC 13812 (PW8)	*C. diphtheriae* (*gravis*)	Yes	44
NCTC 10648	*C. diphtheriae* (*gravis*)	Yes	25
NCTC 10356	*C. diphtheriae* (*belfanti*)	No	106
NCTC 3984	*C. diphtheriae* (*gravis*)	Yes	10
NCTC 5011	*C. diphtheriae* (*intermedius*)	Yes	143
NCTC 13129†	*C. diphtheriae* (*gravis*)	Yes	8
NCTC 12077	*C. ulcerans*	No	NA
ATCC 7715	*C. bovis*	No	NA
ATCC BAA-1293	*C. striatum*	No	NA
6853‡	*C. diphtheriae*	No	395
2337‡	*C. diphtheriae*	No	402
46403§	*C. diphtheriae*	No	391
46337§	*C. diphtheriae*	No	390

*ATCC, American Type Culture Collection; NA, not applicable; NCTC, National Collection of Type Cultures; ST, sequence type. †Clinical isolate from a 72-year-old woman in the United Kingdom isolated following her return from a Baltic cruise in 1997. This isolate is representative of the Russia outbreak clone (*17*). ‡Historical clinical isolates from South Africa ca. 1980s. No clinical information is available for these isolates. §Clinical isolates from 8-year-old (46403) and 9-year-old (46337) endocarditis patients from the Western Cape Province of South Africa, July–August 2015.

### Whole-Genome Sequencing

We extracted DNA from overnight broth cultures with the QIAamp DNA Mini Kit (QIAGEN, Hilden, Germany). We prepared multiplexed paired-end libraries (2 × 300 bp) with the Nextera XT DNA sample preparation kit (Illumina, San Diego, CA, USA) and performed sequencing on an Illumina MiSeq instrument with depth of coverage ranging from 95× to 182×. The raw reads were checked for quality, trimmed, and mapped to the reference genome of *C. diphtheriae* NCTC 13129 (*17*) by using CLC Genomics Workbench version 8.5.1 (CLC Bio-QIAGEN, Aarhus, Denmark), which resulted in an 89.7%–93.8% coverage of the reference genome. We performed de novo assembly for all genomes with CLC Genomics and ordered them relative to NCTC 13129 by using the Mauve genome alignment package version 2.3.1 (*18*). We annotated all genomes by using PROKKA version 1.11 (http://www.vicbioinformatics.com/software.prokka.shtml) and screened the annotated genomes to confirm the presence or absence of the A and B subunits of the *C. diphtheriae* toxin gene and the toxin repressor gene (*dtxR*). *C. diphtheriae* draft genomes for the South Africa isolates have been deposited at DDBJ/European Nucleotide Archive/GenBank (accession nos. MIOA00000000–MIOP00000000, MINX00000000–MINZ00000000, and MIYN00000000–MIYS00000000).

### Multilocus Sequence Typing

We retrieved the sequence type (ST) for each isolate from the whole-genome sequence with the Bio-MLST-MLST-Check module (http://search.cpan.org/dist/Bio-MLST-Check/) and applied the eBURST version 3 algorithm (http://eburst.mlst.net/) to generate a population snapshot for *C. diphtheriae* (*19*). We defined a clonal complex as a cluster of related STs linked as single-locus variants to another ST in the group. We used all available *C. diphtheriae* isolates (n = 616) listed in the global MLST database (https://pubmlst.org/cdiphtheriae/) at the time of analysis (accessed June 13, 2017), including 25 isolates from South Africa, to provide context for the South Africa isolates.

### Whole-Genome Phylogeny and CRISPR Analysis

We constructed the core-genome alignment by using rapid large-scale prokaryote pan genome analysis (Roary) software (*20*) to determine the genetic relatedness between the outbreak, outbreak-associated, historical, and endemic isolates. We generated a maximum-likelihood phylogenetic tree by using RaxML version 8 (*21*). To contextualize the South Africa isolates, we included *C. diphtheriae* genomes from ATCC and NCTC control strains (Table 1) together with publicly available genomes from Brazil (n = 3) (*5*), India (n = 2) (*22*), and Malaysia (n = 2) (*23*) (selected from GenBank). We identified CRISPR-Cas systems in silico with the online CRISPRFinder program (*24*) and determined CRISPR-Cas cassettes by using the classification and nomenclature described by Makarova et al. (*25*).

### Ethics

In South Africa, the National Health Act of 2003 (Act No. 61 of 2003) and the Health Professions Act of 1974 (Act No. 56 of 1974) allow access to patient medical records for those working on investigations directed at ensuring the public health. Further, the Human Research Ethics Committee of the University of the Witwatersrand serves the interests of the public in the collection, analysis, and interpretation of communicable disease data. This institution, which has oversight over the National Institute of Communicable Diseases and ensures the application of good clinical and laboratory practices, approved this outbreak investigation (ethics certification no. M160667). 

## Results

### Description of Patients and Contacts with *C. diphtheriae*

As of June 13, 2015, a total of 15 illnesses were under investigation: 11 were classified as laboratory-confirmed cases and 2 as probable cases (Table 2). One probable case occurred in a patient who was linked to a carrier of toxigenic *C. diphtheriae* and died; the postmortem throat swab from this patient was culture negative. The second probable case occurred in a patient infected with non–toxin-producing *C. diphtheriae* who was linked to 3 carriers colonized with toxin-producing *C. diphtheriae*. The remaining 2 illnesses under investigation could not be classified as confirmed or probable cases; they occurred in culture-negative patients with respiratory diphtheria symptoms who could not be epidemiologically linked to a patient or carrier with toxin-producing *C. diphtheriae*. Of the 11 patients with laboratory-confirmed cases, 6 patients were not up-to-date with the South African vaccination schedule and 2 had received all scheduled vaccines recommended for their age group. Vaccination status was unknown for 4 patients. With the exception of patient 9, who was white, all other patients and carriers were black.

**Table 2:** Characteristics of patients and carriers who were *Corynebacterium diphtheriae* culture-positive, KwaZulu-Natal Province, South Africa, March–June 2015*

No.	Age, y	Isolate no.	Diagnosis	Vaccination status†	Outcome	Specimen type	Biotype	Elek	*tox* gene PCR	ST	CRISPR-Cas system (no. spacers)
1	8	45903	Respiratory diphtheria	Incomplete (missed 18 mo and 6 y boosters)	Died	Tonsillar swab	*mitis*	Pos	Pos	378	I-E-a (33)
2	8	45236	Respiratory diphtheria	Incomplete (missed 18 mo and 6 y boosters)	Survived	Throat swab	*mitis*	Pos	Pos	378	I-E-a (33)
3‡	9	45237	Respiratory diphtheria	Incomplete (missed 6 y booster)	Survived	Throat and nasal swabs	*gravis*	Neg	Neg	395	I-E-a (41), I-E-b (22)
		45238					*mitis*	Pos	Pos	378	I-E-a (37)
4	31	45262	Cutaneous diphtheria	Unknown	Survived	Groin swab	*gravis*	Neg	Neg	395	I-E-a (28), I-E-b (21)
5	9	45902	Respiratory diphtheria§	Unknown	Survived	Tracheal aspirate	*mitis*	Pos	Pos	378	I-E-a (37)
6	5	45463	Respiratory diphtheria§	Unknown	Survived	Tracheal aspirate	*gravis*	Pos	Pos	378	I-E-a (37)
7	8	45461	Carrier§	Unknown	Survived	Throat swab	*gravis*	Pos	Pos	378	I-E-a (37)
8	1	45462	Carrier§	Unknown	Survived	Throat swab	*mitis*	Pos	Pos	378	I-E-a (37)
9	41	45464	Respiratory diphtheria	Unknown	Died	Throat swab	*mitis*	Pos	Pos	378	I-E-a (33)
10	17	45465	Respiratory diphtheria	Incomplete (missed 12 y booster)	Survived	Throat swab	*mitis*	Pos	Pos	378	I-E-a (37)
11	13	45466	Respiratory diphtheria	Incomplete (missed 18 mo, 6 y, and 12 y boosters)	Died	Tonsillar swab	*mitis*	Pos	Pos	378	I-E-a (37)
12	21	45785	Respiratory diphtheria	Unknown	Survived	Throat and nasal swabs	*mitis*	Pos	Pos	378	I-E-a (34)
13	6	45786	Carrier¶	Unknown	Survived	Throat and nasal swabs	*mitis*	Pos	Pos	378	I-E-a (33)
14	11	45784	Probable diphtheria	Up-to-date	Survived	Throat and nasal swabs	*intermedius*	Neg	Neg	395	I-E-a (38), I-E-b (21)
15	11	45789	Carrier#	Unknown	Survived	Throat and nasal swabs	*mitis*	Pos	Pos	378	I-E-a (31)
16	9	45790	Carrier#	Unknown	Survived	Throat and nasal swabs	*mitis*	Pos	Pos	378	I-E-a (31)
17	11	45791	Carrier#	Unknown	Survived	Throat and nasal swabs	*mitis*	Pos	Pos	378	I-E-a (31)
18	10	45792	Carrier#	Unknown	Survived	Throat and nasal swabs	*gravis*	Neg	Neg	395	I-E-a (42), I-E-b (22)
19	13	45787	Respiratory diphtheria**	Incomplete (missed 14 wk,18 mo, and 6 y boosters)	Survived	Throat swab	*intermedius*	Pos	Pos	378	I-E-a (35)
20	4	45788	Respiratory diphtheria**	Up-to-date	Survived	Throat swab	*intermedius*	Pos	Pos	378	I-E-a (35)

*CRISPR, clustered, regularly interspaced, short palindromic repeats; DTaP, diphtheria, tetanus, and pertussis; neg, negative; pos, positive; ST, sequence type.  †The Expanded Programme on Immunization in South Africa recommends DTaP vaccination at 6 weeks, 10 weeks, and 14 weeks with a booster at 18 months, and a tetanus-diphtheria booster at 6 years and 12 years (https://www.health-e.org.za/wp-content/uploads/2014/02/South-Africa-EPI-vaccines-revised-Oct-2010.pdf). ‡Two different genotypes were isolated from 1 patient during the same disease episode. §Members of the same family. ¶Family member of a person who had respiratory diphtheria symptoms and died. The postmortem throat swab from this person was culture negative. #Contact of patient 14. **Members of the same family.

Among the 292 patients and contacts with throat swab samples taken during the KwaZulu-Natal outbreak investigation, we isolated *C. diphtheriae* from 19 persons (Table 2). *C. diphtheriae* isolates from the 11 laboratory-confirmed cases were a mixture of biotypes *mitis* (n = 8), *gravis* (n = 1), and *intermedius* (n = 2). One patient (no. 3) was infected with both toxigenic (biotype *mitis*) and nontoxigenic (biotype *gravis*) *C. diphtheriae*. The 1 culture-positive probable case (no. 14) was defined as such because the patient had respiratory diphtheria symptoms but was culture-positive for nontoxigenic *C. diphtheriae*. However, this patient had 3 contacts who were carriers (nos. 15, 16, and 17) colonized with toxigenic *C. diphtheriae* and 1 contact (no. 18) who was a carrier colonized with non–toxin-producing *C. diphtheriae*. We isolated toxigenic *C. diphtheriae* from carriers (nos. 7 and 8) who were family members of patients with laboratory-confirmed cases (nos. 5 and 6). One carrier (no. 13) had toxigenic *C. diphtheriae* and was a contact of another patient with probable diphtheria who died.

*C. diphtheriae* biotype *mitis* was predominant among carriers (5/7, 71%). Cutaneous *C. diphtheriae* biotype *gravis* was isolated from the groin of a patient (no. 4) who had contact with 2 carriers of non–toxin-producing *C. diphtheriae*. Isolates from both these carriers were discarded at the source laboratory, and no further characterization of their isolates was possible.

### *Tox* and *dtxR* Genes

PCR and whole-genome analysis confirmed the results of the phenotypic Elek testing. In addition, all of the South Africa isolates (both toxigenic and nontoxigenic) were found to harbor an intact *dtxR* gene.

### MLSTs and Population Structure

**Figure 1:**
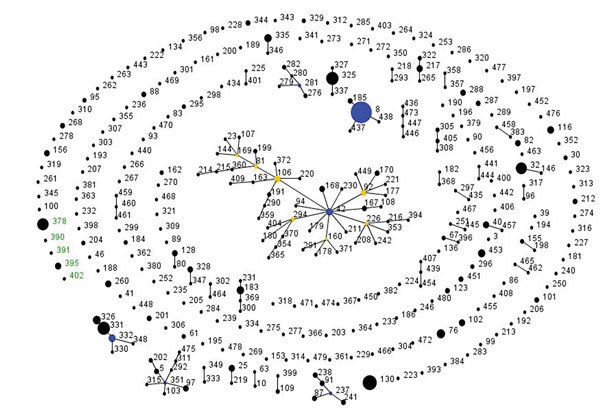
Global population snapshot of *Corynebacterium diphtheriae* sequence types. eBURST (http://eburst.mlst.net/) was used to display the *C. diphtheriae* isolates (n = 616) available in the PubMLST database (http://pubmlst.org/cdiphtheriae/) at the time of analysis (accessed June 13, 2017). Isolates from South Africa (n = 25) are green. The size of each circle is proportional to the number of isolates, and related sequence types are connected by lines. Blue and yellow circles indicate founding and subfounding genotypes, respectively.

At the time of this analysis, the *C. diphtheriae* PubMLST database had records from 32 countries dating from 1948 through 2017, with *C. diphtheriae* isolates from France accounting for 28% of submissions. Overall, the population snapshot revealed a highly diverse population structure for *C. diphtheriae* globally (Figure 1). The 25 South Africa isolates (21 outbreak-associated and 4 historical) represented 5 novel STs: ST-378 (n = 17), ST-395 (n = 5), ST-390 (n = 1), ST-391 (n = 1), and ST-402 (n = 1). The toxigenic outbreak strain from KwaZulu-Natal was ST-378 and the nontoxigenic strain was unrelated ST-395. The 2 historical isolates from the 1980s (both toxin negative) were ST-395 and unrelated ST-402. The toxin-negative isolates from the endocarditis patients were ST-390 and ST-391 (Table 1), each of which shares 4 of 7 alleles with ST-395.

### Core-Genome Phylogeny

**Figure 2:**
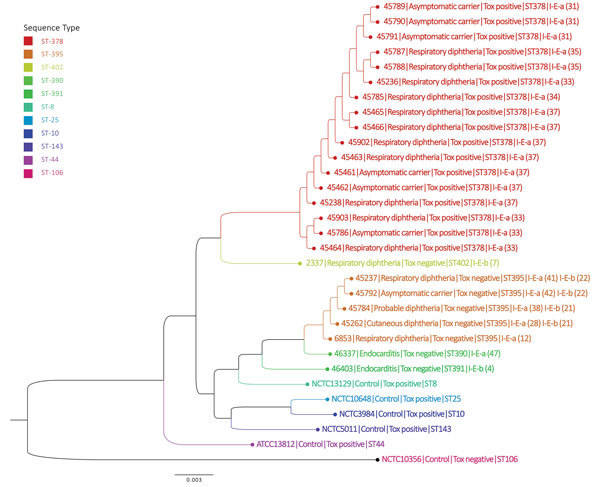
Phylogenetic analysis of *Corynebacterium diphtheriae* isolates based on sequence type, KwaZulu-Natal Province, South Africa, March–June 2015. Maximum likelihood phylogenetic tree demonstrating core-genome phylogeny among isolates from South Africa (n = 25) relative to selected genomes (publicly available from GenBank) from other countries. Scale bar indicates nucleotide substitutions per site. ST, sequence type.

Consistent with MLST data, we identified 2 distinct lineages among the KwaZulu-Natal outbreak isolates. The 17 toxigenic isolates (from 11 patients and 6 contacts) clustered closely together on the whole-genome phylogenetic tree (Table 2; Figure 2). The second lineage consisted of 5 toxin-negative ST-395 *C. diphtheriae* isolates: the isolate from the cutaneous diphtheria patient (no. 4), the isolate from the patient with probable diphtheria (no. 14), an isolate from a carrier (no. 18) linked to the patient with probable diphtheria (no. 14), the nontoxigenic isolate from the patient (no. 3) infected with 2 strains of *C. diphtheriae*, and historical isolate 6853 (Table 1). Comparator genomes from Malaysia (n = 2), Brazil (n = 1), and India (n = 2) and the historical isolate 2337 from South Africa were more closely related to the ST-378 lineage. Two genomes from Brazil (from nontoxigenic isolates) and the UK genome (representative of the Russia outbreak strain) were more closely related to the nontoxigenic ST-395 lineage than to ST-378 (Figure 2; Table 1). The nontoxigenic endocarditis isolates were most closely related to the ST-395 lineage.

### CRISPR-Cas Diversity

All of the toxigenic ST-378 isolates harbored a type I-E-a CRISPR-Cas system, with 5 different variants determined by numbers of spacers (Table 2). The 2 carriers (nos. 7 and 8) and 2 patients (nos. 5 and 6) that were from the same family shared an identical CRISPR-Cas type: I-E-a (37 spacers). This specific variant was present in *C. diphtheriae* from 3 other patients (nos. 3, 10, and 11). The remainder of the ST-378 isolates harbored other type I-E-a variants. Toxigenic *C. diphtheriae* from carriers from the same family (nos. 15, 16, and 17) harbored identical type I-E-a (31 spacers) CRISPRs.

Toxin-negative ST-395 isolates harbored 2 CRISPR-Cas systems, type I-E-a and type I-E-b, and each of the 4 isolates had their own unique combination of variants. Historical isolate 6853 had CRISPR type I-E-a (12 spacers) and historical isolate 2337 had CRISPR type I-E-b (7 spacers), both of which were unrelated to the KwaZulu-Natal isolates. Similarly, *C. diphtheriae* from the 2 endocarditis patients had unique CRISPR types I-E-a (47 spacers) and I-E-b (4 spacers).

## Discussion

We describe the molecular epidemiology of *C. diphtheriae* isolated from a cluster of respiratory diphtheria cases in KwaZulu-Natal, South Africa, during the autumn and winter months of 2015 (*11*). Suboptimal vaccination coverage rates might have contributed to increased vulnerability of older children and adults to *C. diphtheriae* infection, leading to this localized cluster of cases. The KwaZulu-Natal outbreak was caused by a single strain, with a novel ST that does not currently belong to any known clonal complex. This toxigenic strain was unrelated to the nontoxigenic strain isolated during this outbreak investigation from the carrier and patients with respiratory and cutaneous diphtheria. The strain was also not related to the historical and non–outbreak-associated isolates from South Africa or to any other documented *C. diphtheriae* ST from elsewhere in the world.

Biotype does not appear associated with disease severity and is regarded as having limited utility in epidemiologic investigations because of poor discrimination and lack of correlation with genotype (*4*,*26*). Nevertheless, a shift in the predominant circulating biotype of *C. diphtheriae* was demonstrated during a Russia outbreak (*27*,*28*). However, of the 2 studies on the Russia outbreak, neither had genome data available for comparison. In our investigation, no relationship between biotype and genotype was evident. In addition, no epidemiologic correlation with biotype was apparent, as demonstrated by 4 members of the same family being infected with identical *C. diphtheriae* genotypes but having different biotypes (*mitis* and *gravis*).

MLST was consistent with core-genome phylogeny: isolates of the same ST clustered together. In addition to the MLST and core-genome phylogeny, analysis of the CRISPR systems provided additional resolution between the 2 KwaZulu-Natal lineages and allowed a better understanding of the transmission dynamics. Finding *C. diphtheriae* with identical CRISPR variants among family members confirmed circulation and transmission of the same strain. Sequence errors might have resulted in misclassification of some variants that were not confirmed by another method or repeat sequencing; nevertheless, a good depth of sequencing coverage together with other parameters validated the quality of the reads and accuracy of base calling.

*C. diphtheriae* has been shown to exhibit a high degree of genome plasticity (*29*), which was reflected by the highly diverse global population (consisting of mostly unrelated genotypes) seen with the STs from PubMLST. Individual population snapshots by country reiterate this heterogeneity, and many STs are unique to their respective countries (data not shown). One dominant clone ST-42 and its associated single and double-locus variants, which were predominantly isolated in France, accounted for 7% of *C. diphtheriae* isolates in PubMLST; however, the global database is overrepresented by submissions from France and, thus, might not accurately reflect the true population. The MLST database includes a mixture of unrelated STs from countries of North and West Africa, and Angola (the geographically closest neighbor to South Africa for which data were available) has reported a single ST-316 isolate. None of the isolates from Africa share the same or related STs as those identified in the outbreak we report. MLST data for *C. diphtheriae* isolates collected over >20 years (1992–2015) in Algeria showed most isolates were ST-116 (unrelated to any other ST in the PubMLST database), the predominant ST circulating during the 1992–1999 epidemic in that country (*30*). In later years, the population structure of *C. diphtheriae* in Algeria was more heterogeneous. 

The South Africa outbreak-associated isolates represented 2 novel STs not previously reported or related to any other PubMLST-listed ST in the global database. The lack of data for *C. diphtheriae* from other countries in Africa makes it difficult to speculate whether these strains were imported from close neighboring countries or farther away, or whether they are endemic to South Africa. Subsequent to the 2015 cluster of cases in South Africa, we also reported 2 additional cases in May 2016 in the same region, and both were ST-378.

During the KwaZulu-Natal outbreak, co-circulation of the toxigenic and nontoxigenic genotypically unrelated strains were noted during laboratory investigations. Both genotypes (ST-378 and ST-395) were detected in patients and carriers. In 1 case, 2 *C. diphtheriae* isolates (1 toxigenic, 1 nontoxigenic) were detected from the same patient. Because CRISPR analysis indicated that the nontoxigenic isolate had a distinctive combination of CRISPR variants not seen in any of the other isolates, the possibility of contamination was excluded. We expect that disease in this patient was caused by the toxigenic strain and not the nontoxigenic strain. In 1 probable case, a person with symptoms clinically suggestive of diphtheria was infected with the nontoxigenic ST-395 strain but was epidemiologically linked to 3 asymptomatic persons colonized with the ST-378 toxigenic strain. In 1978, a similar phenomenon occurred with a 25-year-old woman in Toronto; 3 different *C. diphtheriae* variants exhibiting 2 different phage types and a mixture of toxigenic and nontoxigenic isolates were isolated from her throat (*31*). In our study, sampling and testing methods might not have been sensitive enough to detect all *C. diphtheriae* variants present. Laboratory workers should be aware of the possibility of mixed *C. diphtheriae* populations in a single person. Although mixtures might be evident through differences in colony and microscopic morphologies, molecular characterization might be more sensitive in detecting such variants. 

Cases of nontoxigenic and cutaneous *C. diphtheriae* might not be clinically as important as toxigenic and respiratory diphtheria and are not notifiable. However, skin lesions can serve as a reservoir of strains that are toxigenic or strains that could potentially become toxigenic if the bacteria possess functional toxin repressor genes and become infected with a *tox* gene–bearing lysogenic corynephage (*32*,*33*). In addition, countries with high vaccination coverage have reported the emergence of invasive disease caused by nontoxigenic *C. diphtheriae*, particularly in high-risk groups such as persons who are homeless, persons who use intravenous drugs or alcohol, persons with diabetes mellitus, and persons with dental caries (*34*–*36*). The South Africa isolates from the 2015 KwaZulu-Natal outbreak were not derived from a single lineage; 2 distinct genotypes were identified within the community. The nontoxigenic lineage has been in circulation for >30 years, verified by sequence typing and close phylogenomic clustering of the archived 1980s isolate (6853) with the ST-395 KwaZulu-Natal outbreak-associated isolates. Because no respiratory or cutaneous diphtheria cases have been reported for several years in South Africa, we have no baseline genotypic data regarding the underlying population diversity, and hence, we are unable to track transmission patterns or changes in genotypes over time. The nontoxigenic South Africa isolates in this study all harbored *dtxR* genes, indicating the potential for toxin production if lysogenized by a bacteriophage.

We identified 2 novel strains of *C. diphtheriae* in a province of South Africa during an outbreak investigation occurring >30 years after diphtheria ceased to be a public health concern. The absence of preexisting molecular sequence data limits conclusions pertaining to the origin of these strains; however, these findings provide baseline genotypic data for future cases and outbreaks as well as information on transmission dynamics for the 2015 outbreak. A better understanding of the molecular epidemiology of this pathogen might assist in directing and strengthening public health interventions. Active and passive surveillance for diphtheria and *C. diphtheriae* carriage is required locally and on the subcontinent, particularly in the context of suboptimal vaccination coverage, especially in older children. We are exploring the idea of a serosurvey in South Africa among different age groups, which might provide insight into the epidemiology of diphtheria in this country.
